# Histoplasmosis Cluster, Golf Course, Canada

**DOI:** 10.3201/eid1201.051083

**Published:** 2006-01

**Authors:** Heather Anderson, Lance Honish, Geoff Taylor, Marcia Johnson, Chrystyna Tovstiuk, Anne Fanning, Gregory Tyrrell, Robert Rennie, Joy Jaipaul, Crystal Sand, Steven Probert

**Affiliations:** * University of Alberta, Edmonton, Alberta, Canada;; † Capital Health, Edmonton, Alberta, Canada;; ‡ Alberta Provincial Laboratory for Public Health, Edmonton, Alberta, Canada;; § National Center for Mycology, Edmonton, Alberta, Canada

**Keywords:** Histoplasmosis, outbreak, epidemiology, soil, dispatch

## Abstract

We report a cluster of 4 cases of acute histoplasmosis (1 culture proven and 3 with positive serology, of which 2 were symptomatic) associated with exposure to soil during a golf course renovation. Patients in western Canada with compatible symptoms should be tested for histoplasmosis, regardless of their travel or exposure history.

Histoplasmosis is endemic in parts of the United States, South America, Southeast Asia, Africa, and Australia (1). Infection is almost always acquired by inhalation from an environmental source, most commonly disturbed earth or bird or bat droppings. Outbreaks and clusters of acute histoplasmosis from common sources are occasionally reported. The distribution of histoplasmosis has been largely described on the basis of these outbreak reports and histoplasmin skin-test surveys (1–5). In Canada, acute histoplasmosis occurs mainly in the central provinces with some evidence of infection in the Atlantic provinces and northern territories (6–10). Sporadic reports of cases from western Canada have been reported; however, local acquisition in these cases could not be verified (11,12). We report a cluster of cases of acute histoplasmosis among persons with no history of travel outside of Alberta during the incubation period for infection. This cluster is associated with exposure to disturbed soil during a golf course renovation.

## The Outbreak

Case 1 (index case-patient): 25-year-old woman with a history of headache, vomiting, fever, and cough sought treatment at a hospital. A chest radiograph showed diffuse nodular pulmonary infiltrates. Computed tomography showed numerous small nodules throughout both lungs. Thorascopic lung biopsy yielded necrotizing granulomata; stains did not show an organism. Histoplasma H and M bands were demonstrated in blood by immunodiffusion (ID) conducted by the National Center for Mycology (Edmonton), and culture of sputum and lung biopsy material yielded Histoplasma capsulatum. Itraconazole therapy was initiated. The patient previously resided in a village in Newfoundland but had relocated to Alberta 5 months before onset of illness. She was a maintenance worker at a golf course in suburban Edmonton and reported that several co-workers had experienced similar symptoms after the renovation of a fairway.

Clinical and exposure information was collected from a core group of 7 persons who worked with the index patient. Three additional probable or possible cases of histoplasmosis were identified and are described as follows.

Case 2: 18-year-old man who was part of the golf course grounds maintenance crew that had worked on the renovation had fever, chills, headache, malaise, chest pain with deep inspiration, anorexia, and fatigue during the same period as the index case-patient, but did not seek medical attention. Blood for serologic testing was drawn 4 weeks after illness onset. The ID test result for H. capsulatum was positive for H and M bands, but a specimen was not submitted to the Centers for Disease Control and Prevention (CDC), Atlanta, Georgia, USA, for complement fixation testing. A chest radiograph demonstrated no abnormalities.

Case 3: 21-year-old man who also worked on the renovation project had fever, chills, nausea, headache, and malaise during the week after onset of the index patient's symptoms; his symptoms gradually resolved over 2 weeks. Serologic testing was conducted. Histoplasma ID was reported as negative, although 1 line of nonidentity was observed. A second sample drawn 6 weeks after illness onset was positive by ID. This specimen was submitted to CDC for further testing; there, ID exhibited both H and M bands and complement fixation testing found a titer of 1:8 for histoplasmin and >1:256 for whole yeast.

Case 4: 37-year-old man who worked in the renovation of the golf course reported no illness during the period of interest. However, as in case 3, despite negative Histoplasma ID results on serologic testing, 1 line of nonidentity was observed. A serum sample drawn from this individual 6 weeks after the index patient's onset of illness was tested at CDC. ID for H. capsulatum was positive for M band only, and complement fixation testing was reported as positive for histoplasmin (titer 1:4) and whole yeast (1:64).

Of the remaining 4 co-workers screened for clinical and exposure information, 1 reported onset of respiratory illness at the same time as the case-patients although all had negative H. capsulatum serology as conducted by the National Center for Mycology. Other golf course employees and contract workers were advised to be tested. Of the 70 staff, 51 provided a serum sample for testing. No additional cases were identified.

Exposure to disturbed soil during golf course renovation was investigated as a possible source of the cluster. Renovations included sod and tree removal, grading of existing soil (no soil was imported), and sod replacement. Workers reported that dust was generated on the course during these activities. Replacement sod came from British Columbia. The approximate incubation periods (i.e., period between the end of the renovation and date of illness onset) for the 3 symptomatic case patients (Cases 1–3) were 6, 6, and 12 days, respectively; however, these are likely underestimates, as the exact date of exposure during the 2-week renovation project is unknown. No accumulated bird or bat droppings within facility buildings nor bird or bat roosting sites on the golf course were evident. Soil samples were tested for H. capsulatum, but all were negative.

## Conclusions

In this cluster of 4 cases, the index case-patient unequivocally experienced acute pulmonary histoplasmosis. Two other persons epidemiologically associated with the index case had compatible symptoms and positive serologic results, and thus can be considered probable case-patients. The fourth patient, although epidemiologically associated with the others, was never symptomatic. While serology from this patient was weakly positive, false-positive serologic results or remote infection cannot be excluded; thus, he is considered a possible case-patient. Together, these cases establish the possibility of local acquisition of histoplasmosis in northern Alberta with a probable micro-focus of soil contamination.

As part of this investigation, an unsuccessful attempt was made to identify soil contaminated with Histoplasma from the implicated golf course. Nevertheless, on the basis of the patients' epidemiologic association and development of symptoms within the incubation time for this infection (13), local soil exposure was the likely source of their infections. A less likely source is the replacement sod imported from British Columbia; however, the laying of sod was not associated with dirt aerosolization and histoplasmosis is not endemic in British Columbia.

The environmental reservoir for H. capsulatum is soil with an acidic pH, some degree of moisture, and moderate temperature. Bird and bat droppings are thought to provide nutrients for H. capsulatum sporulation. Transmission does not occur directly from animals or person-to-person. As inhalation is the nearly universal route of acquisition of this agent, environmental risk factors usually involve the disruption of soil or other infected material (14,15). Aerosolized H. capsulatum can be generated during excavation, construction, or demolition of work areas, cleaning of sites with accumulated bird or bat droppings, and cave treks. Most major urban outbreaks have been associated with earth disruption. Two separate outbreaks in Mason City, Iowa, were related to bulldozing a park and the later removal of trees (15). Data collected in Indiana in the late 1970s and early 1980s identified 2 outbreaks linked to the construction of a tennis complex and excavation for a swimming pool, respectively (15).

This investigation demonstrates the use of acute disease clusters in defining the boundaries of the geographic distribution of histoplasmosis. Before this episode, histoplasmosis has not been considered endemic in northern Alberta; milder, self-resolving clinical cases may have gone unrecognized. Clinicians in western Canada should consider testing for histoplasmosis in clinically compatible patients regardless of their travel or exposure history.
